# Estimating Muscle Activity from the Deformation of a Sequential 3D Point Cloud

**DOI:** 10.3390/jimaging8060168

**Published:** 2022-06-13

**Authors:** Hui Niu, Takahiro Ito, Damien Desclaux, Ko Ayusawa, Yusuke Yoshiyasu, Ryusuke Sagawa, Eiichi Yoshida

**Affiliations:** 1 National Institute of Advanced Industrial Science and Technology, Tsukuba 305-8560, Japan; niu.hui.tp@gmail.com (H.N.); takahiro.ito9@gmail.com (T.I.); k.ayusawa@aist.go.jp (K.A.); yusuke-yoshiyasu@aist.go.jp (Y.Y.); eiichi.yoshida@rs.tus.ac.jp (E.Y.); 2 CNRS-AIST JRL (Joint Robotics Laboratory), IRL, Tsukuba 305-8560, Japan; d.desclaux@laposte.net; 3 Graduate School of Science and Technology, University of Tsukuba, Tsukuba 305-0006, Japan; 4 ISAE-SUPAERO, University of Toulouse, 31055 Toulouse, France; 5 Department of Applied Electronics, Faculty of Advanced Engineering, Tokyo University of Science, Tokyo 125-8585, Japan

**Keywords:** muscle activity, strain, non-rigid registration, point cloud, multilayer perceptron

## Abstract

Estimation of muscle activity is very important as it can be a cue to assess a person’s movements and intentions. If muscle activity states can be obtained through non-contact measurement, through visual measurement systems, for example, muscle activity will provide data support and help for various study fields. In the present paper, we propose a method to predict human muscle activity from skin surface strain. This requires us to obtain a 3D reconstruction model with a high relative accuracy. The problem is that reconstruction errors due to noise on raw data generated in a visual measurement system are inevitable. In particular, the independent noise between each frame on the time series makes it difficult to accurately track the motion. In order to obtain more precise information about the human skin surface, we propose a method that introduces a temporal constraint in the non-rigid registration process. We can achieve more accurate tracking of shape and motion by constraining the point cloud motion over the time series. Using surface strain as input, we build a multilayer perceptron artificial neural network for inferring muscle activity. In the present paper, we investigate simple lower limb movements to train the network. As a result, we successfully achieve the estimation of muscle activity via surface strain.

## 1. Introduction

Assessment of muscle activity is an important topic in the study of human movement. Muscle activity is widely used in the fields of ergonomics, biomechanics, rehabilitation, human motion simulation, and robotics. One of the most common methods of quantifying muscle activity is assessed by electromyography (EMG), which is based on the bioelectrical changes generated during the activity of single or multiple muscle cells or parts of muscle tissue. Electromyography can be guided by electrodes, amplified, recorded, and displayed as a time-series signal graphic. Quantifying muscle activity using EMG is a contact measurement method, which requires the installation of additional sensors and has strict requirements on the test environment, meaning that the use of this type of method has many limitations. Therefore, we want to find a non-contact way to obtain muscle information that is suitable for various situations.

Non-contact visual measurement can be a good solution to this problem. Some visual features can be considered cues to estimate a person’s muscle activity, for example, joint position and skin surface deformation. While many studies have assessed muscle motion status by recording and extrapolating joint trajectories, the present study focuses on investigating the relationship between muscle activity and skin surface deformation. This requires that we have the ability to obtain a more detailed model of motion through the visual system. Physical information, such as muscle force, can be estimated from the deformation of skin by a visual sensing system, as reported in [1,2]. If the deformation of an object is hard to explain by a small number of parameters, then it is necessary to observe many points on the object simultaneously in order to obtain sufficient information of deformation. Visual observation by a camera is suitable for this purpose. Various approaches have been proposed. One major method is to compute the 3D shape by a range sensor and calculate the 3D shape by finding the corresponding points between consecutive frames. The shape of a deforming object can be obtained using RGBD cameras [3,4]. Since these cameras output range data at 20–60 frames/s, the shape of a moving object at each moment is captured frame by frame. If the shape is rigid and has shape features for unique correspondence, the motion is given by a method of matching shapes, such as the iterative closest point (ICP) method [5].

One of the problems in using range data for physical analysis of a human body is noise in the raw range data. Whether a range sensor is based on triangulation or time-of-flight (ToF), depth noise occurs due to the error of finding correspondence in a stereo system or the error of calculating the time duration between light emission and detection. Since the range data are obtained frame by frame and depth noise is independent of the deformation, the motion estimated by deformable registration between two consecutive frames is affected by depth noise. The trajectory of deformation can be vibratory in some cases, which degrades the physical analysis of the target. In the present paper, we propose to use temporal constraints between range data in order to reduce the registration error induced by the noise. By combining the constraint given by temporal sequence and texture information, the proposed method allows for tracking points more accurately. The registration with the new constraints can estimate deformation along both in-surface and out-of-surface directions.

In addition, using only shape features is, however, insufficient to find correct correspondences in the case of a deformable object because shape features are largely affected by deformation. As a solution to solve this problem, the proposed approach consists of using a deformable ICP with a texture correspondence. The use of this additional color information allows the avoidance of surface slipping and improvement of the results in the case of complex tangential movement along the model surface. We introduce a surface strain tensor to represent the skin surface deformation and try to build a model for inferring muscle activity using deep learning methods.

To express the deformation, the displacement of each vertex from a template shape is a candidate as used in [1]. The problem with the previous method is that it is necessary to align each bone to obtain the coordinates for calculating the displacement. Since the bones need to be aligned by the registration of deformed surfaces, it will be unstable if the deformation is large. To overcome this disadvantage, we propose a method to express the deformation by strain. Additionally, because the relationship between strain and muscle activity has many factors, including muscle amount, fat under skin, and joint angle, it is difficult to explain by simple expression. That is the reason we take a learning-based approach that can express a nonlinear relationship.

## 2. Related Research

Human motion models are extremely complex, and, in general, there is a tendency to use simplified models to describe motion in human motion simulation. This includes the calculation of human inertia parameters: methods based on forward dynamics, and methods based on inverse dynamics. Either of these methods is a reasonable abstraction of the human body model, considering various complex physical situations to ensure the physical reality of the model. However, it is still a challenge to verify the correctness of the simulation results.

The use of electromyography to evaluate muscle movement is also a more conventional method. Surface electromyography (sEMG) [6] is an easily detectable neural signal of the human body that is rich in human movement information. It is possible to analyze the relationship between EMG signals and muscle forces and corresponding joint moments using EMG signals as input signals and combining these signals with relevant biological models for a deeper understanding of human dynamics. In recent years, many authors have proposed methods for solving joint moments using EMG. The models used are, for example, Huxley’s more complex biophysical model [7] and Hill’s classical model [8,9]. Electromyography signals can be used to measure muscle activation. However, the mapping relationship between the EMG signals and muscle activation is highly nonlinear, and the amplitude of the EMG signal is affected by many factors. Generally, we consider the root mean square (RMS), integrated electromyography (iEMG), or averaged electromyography (AEMG) to reflect local muscle movements. A previous article [10] gives an introduction to expressing muscle activity using iEMG. For the reconstruction of scenes, 3D reconstruction by depth sensors is a common operation. This technique has been studied intensively, especially for rigid objects [5,11]. Along with the development of RGBD sensors, range data captured at high frame rates are now easily accessible. Registration of range data of a deforming object has become important to analyze the characteristics of the object.

In 3D deformable registration, the transformation for each vertex of a point cloud is estimated. The local features of shape and texture are used to find the corresponding point and calculate the transformation. However, since the local feature is often not sufficient to determine the transformation, regularization of transformation is required. Two types of regularization can be considered: spatial and temporal constraints.

In the case that two point clouds are registered, the spatial constraint between neighboring points is used. Various spatial constraints have been studied, such as isometric mapping [12,13], as-rigid-as-possible [14,15,16,17,18], membrane model [19], and conformal mapping [20,21,22]. Spatial constraints are also used in the case of registering multiple frames [23,24]. In the present paper, the proposed method uses a spatial constraint based on conformal mapping because this constraint is applicable to various deformations, as compared to other constraints. If more than two point clouds captured sequentially are considered, assuming plausible motion at each vertex can provide useful information as a temporal constraint. Temporal flicker of range data is reduced during merging shapes of multiple frames in [25,26]. These methods remove the noise at each vertex after registration, and the correspondence for taking the average is calculated before removing noise. Noise removal, however, should be performed simultaneously or before finding correspondence.

Some methods that consider a temporal constraint during registration have been proposed, including averaging vertex positions of volumetric data with the prior motion as a temporal force [27], registration with local deformation along the line of sight [28], and minimization of the magnitude of acceleration as a temporal constraint during registering three consecutive frames [29]. These methods consider noise to find the correspondence between multiple range scans in a batch procedure. If many range scans captured by an RGBD sensor need to be registered, a filtering approach in which a pair of range scans are registered sequentially is appropriate and feasible to reduce the computational cost.

Another approach is focused on the deformation of a whole body by extending [26]. The human skeleton is introduced as a priori information to achieve robust dynamic reconstruction of the human body. The vertices are assigned to bones, and the motion is constrained by the motion of bones. The positions of the bones are temporally averaged [30] or the temporal difference is minimized from the previous frame [31]. These assumptions improve robustness for rough estimation of body parts, but are not sufficient to remove the flicker of each vertex.

Since finding accurate correspondences for a deforming object only by shape features is difficult, using the color and texture to improve the process became more usual. Some methods introduced color information in the ICP scheme [32,33,34]. Since one of the problems is to reach a local minimum in optimization, approaches to avoid local minima were proposed in later studies.

Using weighted fiducial markers was proposed in order to improve the results of the registration in [35]. Transformation to a conformal plane in order to search for correspondence in the texture was used in [36]. These approaches have accuracy limits due to relying on few points or the requirements of good assumption on the shape. Another approach is to use the error defined by photometric and geometric error based on local color gradient [37]. However, the limitation is that this requires dense textured models and good registration results. The proposed approach assumes sparse texture features, which are easy to detect and more robust as compared to dense features, to find corresponding features. Even if the positions of corresponding features are not very close to each other, the correct correspondence can be found. In addition, if tangential motion on an object’s surface is smooth like human skin, the motion can be interpolated from the motion of sparse features.

## 3. Proposed Method

### 3.1. Data Acquisition

Considering the complexity of muscle movements near joints with a large degree of freedom, such as the shoulder and near the elbow, we consider studying the relatively simple lower limb. We studied the flexion and extension movements of the ankle joint, focusing on two muscles, namely, the gastrocnemius and the soleus. We used data from previous studies in our experiments [1].

**Figure 1:**
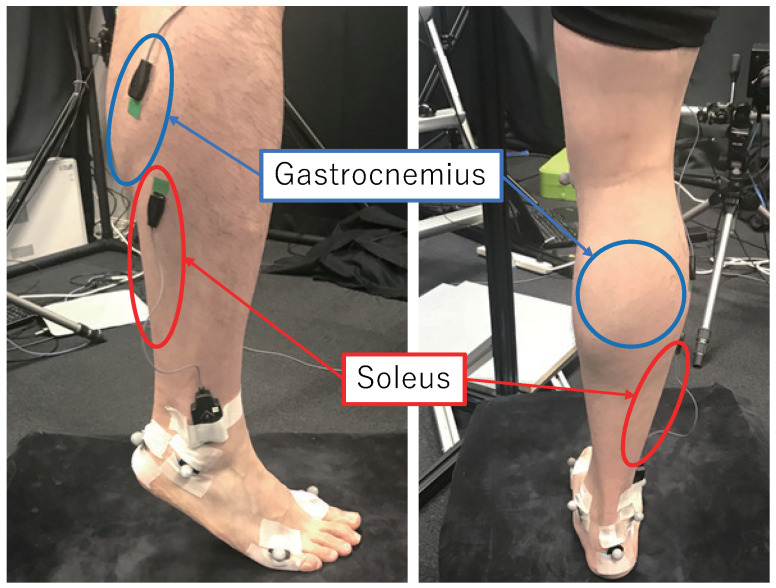
Setup. An electromyography (EMG) sensor is placed on the outer side of right leg. The skin shape of the lower limb is observed using a three-range-sensor system.

In order to obtain a 3D model of the leg, a system of range sensors based on a projector-camera system was constructed in a previous study [38]. Three sensors were configured to capture the shape, and the experimental method was based on that of a previous study [39]. The experimental setup is shown in Figure 1.

**Figure 2:**
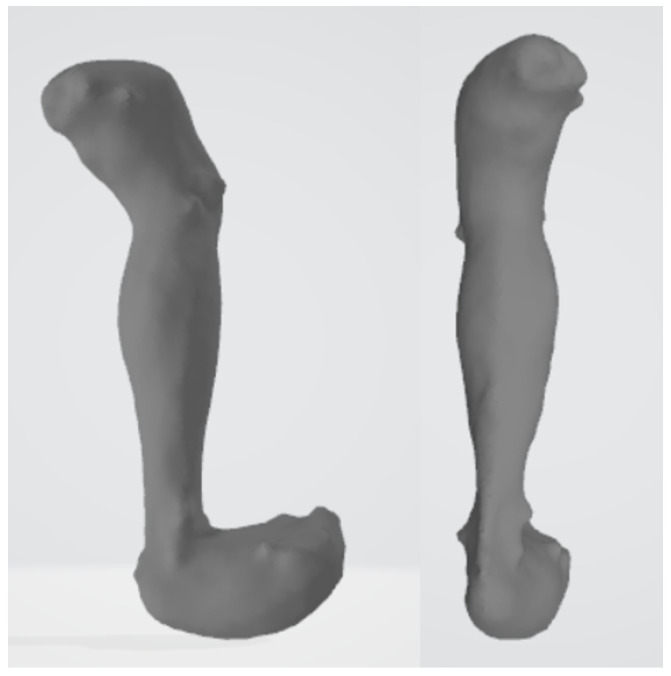
Sample of skin shape model after Poisson reconstruction.

The final model is obtained by combining the shapes captured by the depth camera system via Poisson reconstruction [40]. The shape of the EMG sensors on the skin is removed from the range scans, and the skin shape is interpolated during their merger. Here, Figure 2 shows a sample of the acquired skin shape model.

The muscle activity data were calculated from the measurements of the EMG sensors, which were arranged on the gastrocnemius and the soleus. The muscle activity $A_{k}$ at each frame is defined as the integrated EMG signal normalized by the signal of the maximal voluntary contraction (MVC) [10].

(1) $$\begin{array}{cc} a_{k}= & a_{k-1}+\Delta t(\sum_{j=k-n}^{k-1}|e_{k}|-a_{k-1})/D \end{array}$$

(2) $$\begin{array}{cc} A_{k}= & a_{k}/a_{\mathrm{MVC}}, \end{array}$$

where $e_{k}$ is an EMG value at the *k*-th frame, Δt is a timestep, and *D* is a parameter, which is 0.04 if $i_{k}>a_{k-1}$ and 0.07 otherwise. *n* is a user defined parameter and n=3 in this paper. $a_{\mathrm{MVC}}$ is the activity of MVC. Refer to [9] for the details.

**Figure 3:**
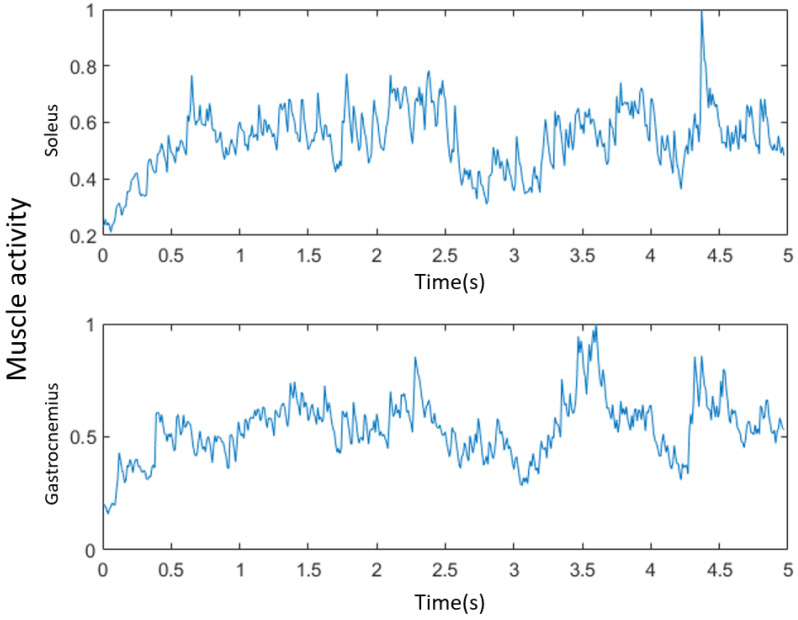
Muscle activity calculated from a set of EMG data.

A set of muscle activity calculation results is shown in Figure 3.

### 3.2. Non-Rigid Registration

After acquiring the 3D reconstruction data, we proceed to the registration step. We consider the provided template as a mesh, which is composed of point clouds. In order to match the template to the target, we deform the template so that the distance between the corresponding points is minimized. In order to estimate this deformation, we consider minimizing the energy parameter given to each corresponding vertex. In this method, we define the cost function *E* as follows:

(3) $$\begin{array}{c} E=w_{C}E_{C}+w_{F}E_{F}+E_{R}, \end{array}$$

where $E_{C}$ is the geometrical error between the closest point, $E_{F}$ is the geometrical error between corresponding feature points, $E_{R}$ is the constraint to avoid extreme local deformation, and $w_{C}$ and $w_{F}$ are the weights as defined by users. This error is optimized based on an iterative closest point (ICP) scheme loop. First, the correspondence between points is found between template and target surfaces. Second, the optimal transformation for each template vertex is estimated by reducing the error. These two steps are repeated until reaching the desired accuracy.

The $E_{R}$ term contains spatial and temporal constraints:

(4) $$\begin{array}{c} E_{R}=w_{S}E_{S}+w_{T}E_{T}. \end{array}$$

The term $E_{S}$ describes the spatial constraint in the proposed method based on as-conformal-as-possible (ACAP) non-rigid registration [22], as defined later in Equation (7). Here, $E_{T}$ is a new term introduced in the present study that imposes a temporal constraint to register a sequence of range scans, which will be described hereinafter. The term $E_{C}$ is calculated based on the ICP by finding the closest point of the target surface for each template vertex. Here, $E_{F}$ is a term related to the information contained in the texture, which is defined by color marker error. This error is a geometrical error between color markers matched from the template mesh to the target mesh. Three-dimensional registration methods based on the ICP use the closest point for each template vertex as the correspondence. Methods by which to find the closest point have been studied in the literature [41]. In the present paper, the proposed method finds the closest point based on a nearest neighbor search, which means point-to-point correspondence between template and target vertices.

**Figure 4:**
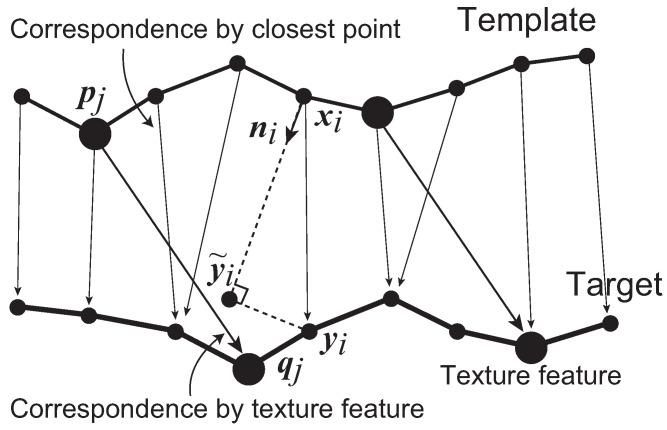
Two-dimensional slice of a situation of the corresponding points.

Let $x_{i}$ and $y_{ik}$ be the homogeneous coordinates of the corresponding vertices of the current template and target surfaces at the *k*-th frame. The deformation is expressed as a set of 3×4 affine transformation matrices $X_{ik}=\left[T_{ik};t_{ik}\right]\left[R_{rk};t_{rk}\right]$ that are associated with each *i*-th vertex of the template, where $T_{ik}$ is a linear transformation, and $t_{ik}$ is a translation. Moreover, $R_{rk}$ and $t_{rk}$ are a rotation matrix and a translation vector, respectively, that are common for all vertices for the *k*-th time frame. Therefore, it is assumed that the transformation can be represented by global rigid transformation and local deformation. The energy by the closest points is defined as follows:

(5) $$\begin{array}{c} E_{C}=\sum_{i}{∥X_{ik}x_{i}-{\tilde{y}}_{ik}∥}^{2}, \end{array}$$

where ${\tilde{y}}_{ik}=x_{i}+\left(R_{rk-1}n_{i}\cdot \left(y_{ik}-x_{i}\right)\right)R_{rk-1}n_{i}$, and $R_{rk-1}$ is the global rotation of the previous frame. In order to avoid the large deformation along the tangential directions, the energy is calculated by projecting the displacement to the normal vector $n_{i}$ of template $x_{i}$. Figure 4 shows a 2D slice of the situation where $p_{j}$ denotes the *j*-th texture feature on the template, and $q_{j}$ is the corresponding texture feature on the target. The corresponding point is given as the closest vertex for this energy. Since vector $y_{ik}-x_{i}$ is perpendicular to the template surface, this energy is used for the deformation along the out-of-surface direction.

**Figure 5:**
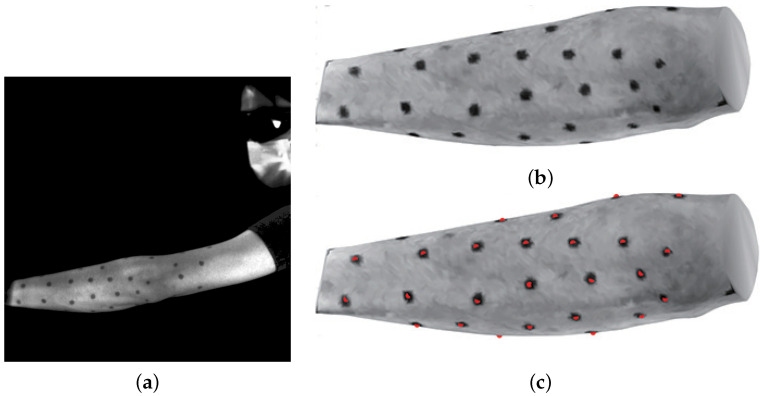
Markers attached to a forearm are detected on a template mesh. (**a**) Input image. (**b**) Template mesh. (**c**) Detected markers.

Texture features used for registration consist of dot markers, detected by the color difference of the vertices compared to neighboring vertices. Figure 5a is an image of observing an arm, and Figure 5b is the template mesh of the forearm with dot markers. As proposed previously [35], the color difference from the vertices in the first neighboring ring of each vertex is calculated. The groups of vertices that have large differences are detected, and vertices that are closest to the mean positions are chosen as the positions of the dot markers. The red points in Figure 5c indicate the detected position of the markers.

Since we consider that the frame rate is rather high compared to the displacement speed of the shape, it can be assumed that the distance of texture features between the target and the template mesh is small. Moreover, in most cases, the deformation is slower than the global transformation of the shape, especially in the case of the human body. Based on these assumptions, a simple closest point matching is sufficient and more reliable than other methods, such as gradient-based methods, in most cases. Since a false positive matching will degrade the accuracy, the proposed method uses thresholding about the maximal distance for matching, based on the median value of the matching distance from the previous step. Once matching of texture features is given, the error is defined as follows:

(6) $$\begin{array}{c} E_{F}=\sum_{j}{∥X_{j}p_{j}-q_{j}∥}^{2}, \end{array}$$

where $p_{j}$ is a vertex of the template detected as a marker, and $q_{j}$ is the target marker corresponding to $p_{j}$. This energy is used for the deformation along the in-surface direction by finding the sliding motion of the surface by matching texture features.

Since we consider that the frame rate is rather high compared to the displacement speed of the shape, it can be assumed that the distance of texture features between the target and the template mesh is small. Moreover, in most cases, the deformation is slower than the global transformation of the shape, especially in the case of the human body. Based on these assumptions, a simple closest point matching is sufficient and more reliable than other methods, such as gradient-based methods, in most cases. Once matching of texture features is given, the error is defined as follows.

The spatial constraint consists of three components: as-conformal-as-possible, consistency, and smoothness terms:

(7) $$\begin{array}{c} E_{S}=E_{ACAP}+E_{consist}+E_{smooth}. \end{array}$$

Here, $E_{ACAP}$ penalizes if $T_{ik}$ differs from a rotation matrix with scaling. Moreover, $E_{consist}$ penalizes inconsistencies between linear transformation and translation of one-ring neighbors of each vertex to make the problem well posed, and $E_{smooth}$ penalizes inconsistencies of transformation between a vertex and one-ring neighbors to avoid local extreme deformation. Refer to [22] for details.

In order to introduce the temporal regularization, let us define the velocity and acceleration of each vertex on the template. In the registration process at each frame, the position, velocity, and acceleration of each vertex at the previous frame are considered to be known. Let $\left[T_{ik-1};t_{ik-1}\right]$, $\left[{\dot{T}}_{ik-1};{\dot{t}}_{ik-1}\right]$, and $\left[{\ddot{T}}_{ik-1};{\ddot{t}}_{ik-1}\right]$ be the affine transformation and its time derivatives for each *i*-th vertex at the previous (k−1)-th frame. The current state can be computed as follows:

(8) $$\begin{array}{c} \left[T_{ik}\;t_{ik}\right]=[T_{ik-1}\;t_{ik-1}]+\Delta T[{\dot{T}}_{ik-1}\;{\dot{t}}_{ik-1}]\\ \;+\frac{1}{2}\Delta T^{2}\left(\left(1-\beta \right)[{\ddot{T}}_{ik-1}\;{\ddot{t}}_{ik-1}]+\beta [{\ddot{T}}_{ik}\;{\ddot{t}}_{ik}]\right) \end{array}$$

(9) $$\begin{array}{c} \left[{\dot{T}}_{ik}\;{\dot{t}}_{ik}\right]=[T_{ik-1}\;t_{ik-1}]\\ \;\;+\Delta T\left(\left(1-\gamma \right)[{\ddot{T}}_{ik-1}\;{\ddot{t}}_{ik-1}]+\gamma [{\ddot{T}}_{ik}\;{\ddot{t}}_{ik}]]\right) \end{array}$$

where $\left[{\dot{T}}_{ik};{\dot{t}}_{ik}\right]$ and $\left[{\ddot{T}}_{ik};{\ddot{t}}_{ik}\right]$ are the velocity and acceleration, respectively, at the current *k*-th frame, and ΔT is the time step of the data sequence. The current state is computed by the Newmark-β method with the scalar parameters β and γ. The feature of this implicit time integration method is to express the current local affine transformation $\left[T_{ik};t_{ik}\right]$ and its velocity $\left[T_{ik};{\dot{t}}_{ik}\right]$ using not only the previous acceleration $\left[{\ddot{T}}_{ik-1};{\ddot{t}}_{ik-1}\right]$, but also the current acceleration [${\ddot{T}}_{ik};{\ddot{t}}_{ik}]$, which lead to the numerical stability of the time integration along the time sequence. The term of the temporal regularization is represented as follows:

(10) $$\begin{array}{c} E_{T}=\sum_{i}\left({∥{\ddot{t}}_{ik}∥}^{2}+\omega_{T}\mathrm{tr}\left({{\ddot{T}}_{ik}}^{T}{\ddot{T}}_{ik}\right)\right)\\ +w_{v}\sum_{i}\left({∥{\dot{t}}_{ik}∥}^{2}+\omega_{T}\mathrm{tr}\left({{\dot{T}}_{ik}}^{T}{\dot{T}}_{ik}\right)\right) \end{array}$$

where $w_{v}$ and $w_{T}$ are the coefficients for the evaluation term regarding the velocity and the linear transformation, respectively. Note that $\left[T_{ik};t_{ik}\right]$ and $\left[{\dot{T}}_{ik};{\dot{t}}_{ik}\right]$ are represented by $\left[{\ddot{T}}_{ik};{\ddot{t}}_{ik}\right]$, as shown in functions 6 and 7. Therefore, the optimization needs to be solved with respect to $\left[{\ddot{T}}_{ik};{\ddot{t}}_{ik}\right]$ instead of $\left[T_{ik};t_{ik}\right]$. The above temporal regularization only reduces the high velocities and accelerations related to local non-rigid deformation. Note that the temporal regularization can be also considered in the registration when estimating the global rigid transformation $\left[R_{rk};t_{rk}\right]$. Such a problem is equivalent to the trajectory optimization of multi-body systems [42].

Before the non-rigid registration step, a rigid registration is applied to estimate the global transformation for each frame. The following algorithm is then applied to each frame of the target mesh. The result of the previous frame is used for initialization and time integration. The parameters are set as identity matrices and zero vectors in the first frame. The procedure of the proposed methods is summarized as follows.

1. Initialization.
2. Detect texture markers of the target and find matches of each marker on the template.
3. Apply global rigid transformation based on rigid ICP using the $E_{C}$ and $E_{F}$ terms.
4. Iterate the following steps of deformable registration until convergence.
  (a) Update the closest point in the target for each vertex of the template.
  (b) Calculate *E* and estimate the acceleration of the transformation parameters of each template vertex by minimizing the energy.
  (c) Calculate the position and velocity of the current frame by integrating the acceleration.

### 3.3. Computation of Strain Tensor

**Figure 6:**
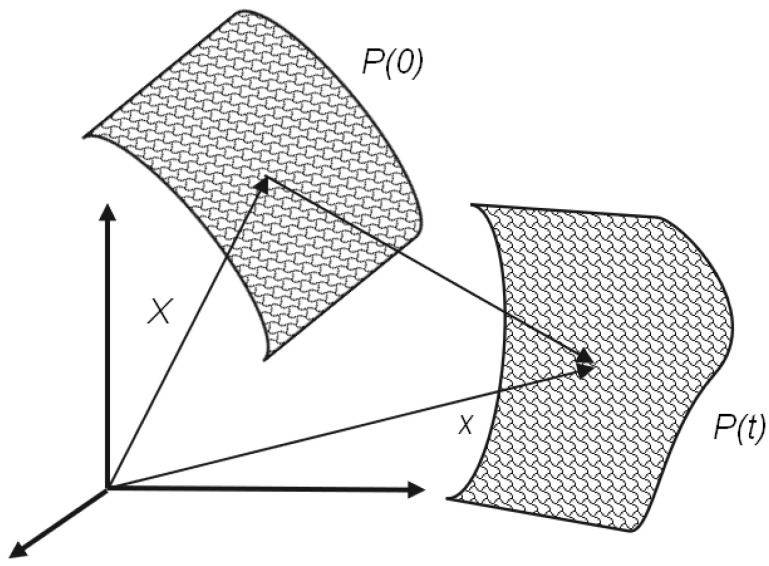
In this figure, *X* is mapped to the new point, *x*, after the deformation.

Next, we compute the strain tensor from the surface of the registration results. In order to describe the deformation from a base configuration to a reference configuration, as shown in Figure 6, or from the reference configuration to the current configuration, we need to define some terms for deformation measures.

The tensor is characterized by means of gradients of motion. The partial division of current displacement related to the current location and reference location arranged in a Jacobean format is called the deformation gradient matrix, which is represented as follows:

(11) $$F=[\begin{array}{ccc} \frac{\partial \left(x\right)}{\partial \left(X\right)} & \frac{\partial \left(x\right)}{\partial \left(Y\right)} & \frac{\partial \left(x\right)}{\partial \left(Z\right)}\\ \frac{\partial \left(y\right)}{\partial \left(X\right)} & \frac{\partial \left(y\right)}{\partial \left(Y\right)} & \frac{\partial \left(y\right)}{\partial \left(Z\right)}\\ \frac{\partial \left(z\right)}{\partial \left(X\right)} & \frac{\partial \left(z\right)}{\partial \left(Y\right)} & \frac{\partial \left(z\right)}{\partial \left(Z\right)} \end{array}]$$

In the present paper, we use the Green–Lagrangian strain tensor defined as follows:

(12) $$\begin{array}{c} E_{ik}=\frac{1}{2}\left(F_{ik}^{t}F_{ik}-I\right), \end{array}$$

where *I* is the identity matrix and $F_{ik}$ is the deformation gradient tensor that represents the gradient of the mapping from the reference vertex position to the current vertex position. In this experiment, we simply estimate $F_{ik}$ by computing a regression matrix from the reference space to the current space. Both spaces are represented by several neighbor vertices of the *i*-th vertex that are chosen by distance thresholding. After computing strain tensor $E_{ik}$, we obtain the first invariant of $E_{ik}$ that represents the sum of the principal strains as follows:

(13) $$\begin{array}{c} I_{ik}=tr\left(E_{ik}\right). \end{array}$$

We use $I_{ik}$ as a measure of the strain of the surface. A heat map of the strain generated by $I_{ik}$ is used to visualize the area in which the shape is deformed.

### 3.4. Predicting Muscle Activity

**Figure 7:**
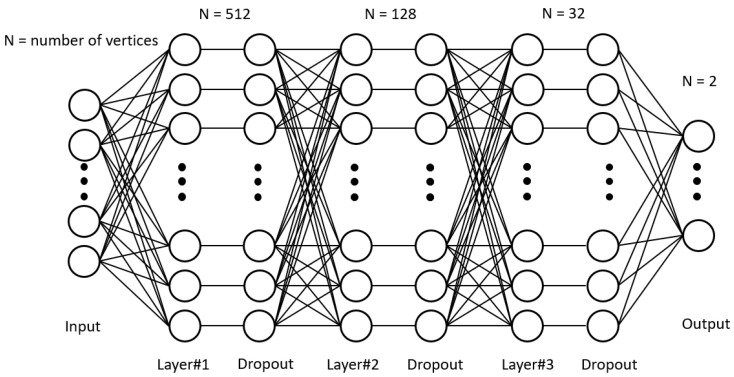
Structure of the network.

As mentioned above, muscle movement is a relatively complex system, and artificial neural networks have the ability to learn the complex relationships between features and targets. We therefore consider a network to accomplish muscle motion prediction in a nonlinear manner. In order to implement a neural network for regression, the architecture of the neural network must be defined. As shown in Figure 7, the neural network uses a simple multilayer perceptron (MLP) to define the architecture.

We presuppose the problem as a higher-order nonlinear regression problem. The MLP can be seen as creating a matrix that can be learned, where each dimension of the output is obtained by weighting the dimensions of each input, with an activation function added in between, allowing the model to learn nonlinear relationships. The MLP has a large number of parameters, which corresponds to providing a strong representation that can approximate any function. This makes it reasonable to think about the regression of the problem using MLP. We take as input the strain information of all vertices of the point cloud for each frame, and the corresponding output is the activity of the two muscles (obtained from the EMG information in the previous section). Note that, here, the order of arrangement does not represent the spatial relationship of the point cloud. Several key points for the construction of the MLP machine are weight initialization, the choice of activation function, the use of back propagation, the choice of earning rate and loss function, and regularization. Initialization usually uses the Gaussian distribution random initialization method to assign weights to prevent layer activation output from exploding or disappearing during the forward transmission of deep neural networks. For optimization, we use the stochastic gradient descent (SGD) approach. Each iteration calculates the gradient of the mini-batch and then updates the parameters. For calculation of the loss, we use the mean squared error (MSE), which is commonly used in regression models. The regularization method is a strategy by which to reduce the variance, which is commonly understood as mitigating the phenomenon of over-fitting. In the present study, we applied the dropout for random deactivation.

Since the small sample and the deep network parameters are too huge to affect the amount of computation, we build a relatively light network containing three hidden layers. All frames of an action are treated as a batch, and each layer uses Relu as the activation function, using random deactivation to complete the regularization part.

## 4. Experiments

In this section, we perform two experiments. In the first experiment, we want to evaluate whether a registration that introduces a time-constrained term can better help the motion to be tracked. We evaluate the proposed method with both the simulation model and captured point cloud data. In the second experiment, we define the prediction of muscle activity using a deep learning network.

### 4.1. Evaluation of Registration

The goal of the proposed method is to estimate muscle activity, and the model is created based on the muscle model [43] to simulate the deformation of the muscle according to the activity. Although the real human body consists of various other components, including bones, fat, and skin, the model is simplified and consists of muscle fibers. The accuracy of the proposed deformable registration is evaluated using the simulated model. We also apply the method to the motion tracking of the human arm and leg. Since the proposed method is designed for motion tracking and muscle activity analysis, we show that the resultant information of the vertex is reliable for these analyses. The data involving human subjects were obtained with the approval of the AIST Committee for Ergonomic Experiments.

#### 4.1.1. Evaluation Using Ground-Truth Simulated Deformation

First, the noise tolerance of the proposed registration method is evaluated. We test both the proposed method and the method without temporal constraint by using the muscle model. In the simulation, the model gradually expands according to muscle activity. The target meshes are created by adding random noises to the time-sampled data. The length of the model is 120 mm, and the shapes are sampled at 100 Hz for one second. The simulation model without noise at the first frame is used as a template mesh, and the proposed method is applied to deform the template to fit the target mesh at each frame.

**Figure 8:**
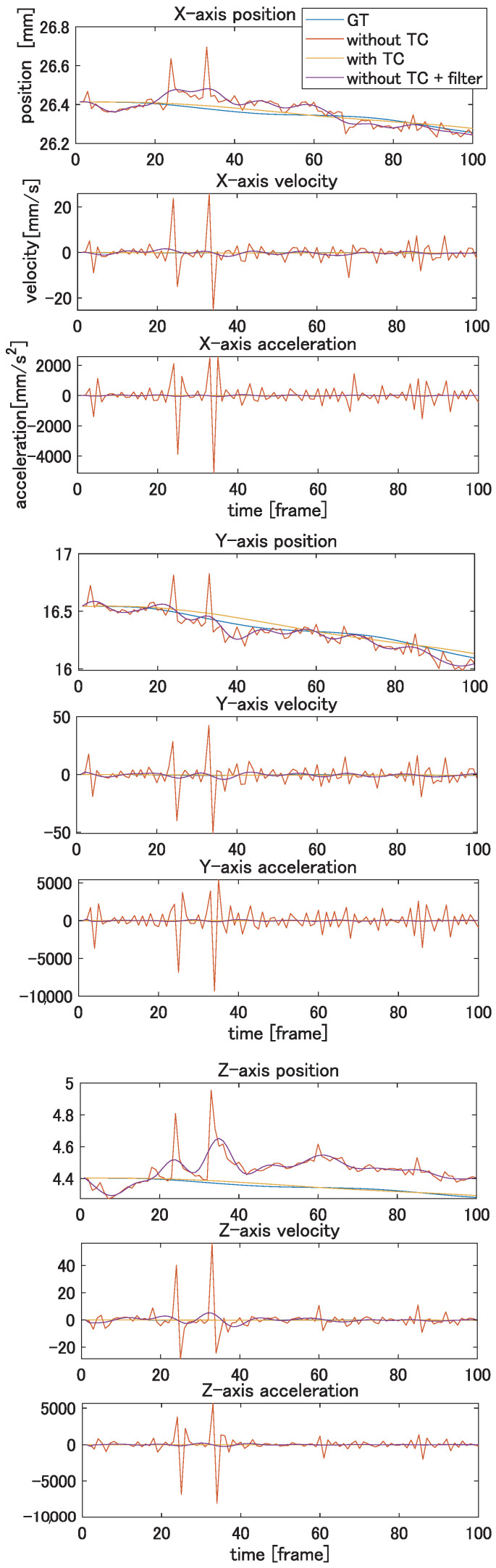
Results of tracking simulation model. Upper row: position, middle row: velocity, and lower row: acceleration in each axis.

In this experiment, we evaluate the effectiveness of the temporal constraint (TC) introduced in the present paper. The proposed method is compared with the result without a TC. Since a temporal filter can be applied to the registration result as an option to reduce flicker, the second method for comparison is temporal filtering after registration without a TC. In this experiment, a seventh-order low-pass Butterworth filter with a cut-off frequency of 10 Hz is applied to the results without a TC. Figure 8 shows the results of tracking the vertex of the model during motion. Each column corresponds to an individual axis, and rows correspond to the position, velocity, and acceleration. In this figure, the trajectory of one of the vertices in the area with the highest shape deformation is plotted. As shown in Figure 8, the proposed method can reduce the noises. In particular, the proposed method can significantly improve the accuracy of velocity and acceleration, which are important for further analysis of the dynamic movement of the target object. Table 1 shows the registration errors of these three methods. The errors are calculated as the mean squared error from the ground-truth value of all vertices of all frames. The proposed method with a TC shows the best accuracy for all metrics of position, velocity, and acceleration. If the shapes are registered without a TC, the velocity and acceleration become much worse than that with a TC, as expected. The proposed method shows better results, even if temporal filtering is applied after registration.

**Figure 9:**
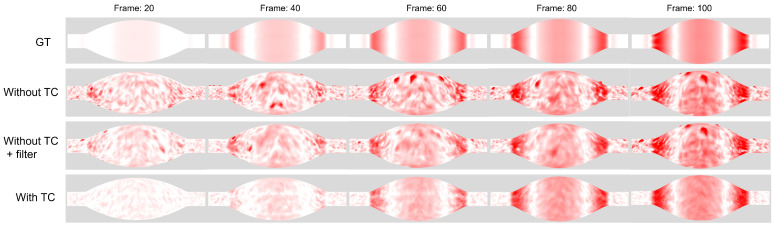
Heat map of strain from the registration results of the simulation model.

Based on computing the magnitude of strain by Equation (13), the heat map of the strain is obtained as shown in Figure 9. The magnitude of the strain is indicated by the gradation of the color from white to red (red indicates higher value). The first row shows the heat map created from the registration result of the ground-truth target, the second one shows the registration without the TC of the noise target, the third one shows the registration without TC and applied filter, and the last rows shows the registration with TC. Figure 9 shows that our method significantly reduces the noise and achieves better estimation of the strain heat map. The mean absolute percentage error (MAPE) of the strain magnitude for all vertices of all frames compared to the ground truth is shown in Table 2.

#### 4.1.2. Tracking Real 3D Scans

**Figure 10:**
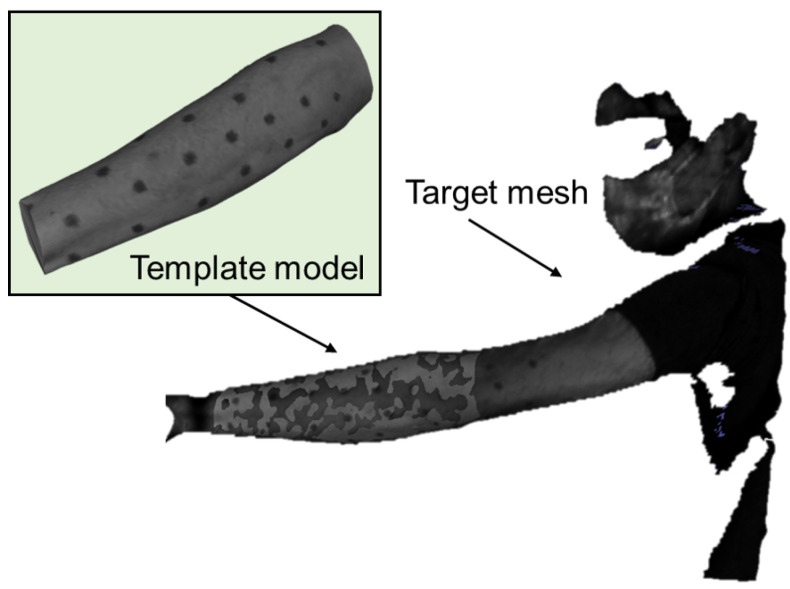
Reconstruction of arm twisting motion.

Next, we evaluate the proposed method with the point clouds that are obtained by capturing human skin. In the experiments, the point clouds are captured by using a previous method [38] at 1000 FPS in combination with another previously reported method [39] to obtain the texture. The texture is monochrome in the experiments. Figure 10 shows an example of 3D acquisition that corresponds to the image in Figure 5a.

In order to clarify the contribution of the proposed method, the parameters for 3D reconstruction are set in order to obtain noisy results. Figure 10 also shows the template mesh that is obtained by capturing a fixed shape of the target from various directions and merging the meshes into a single mesh using a Creaform handy 3D scanner.

In this experiment, we capture the arm twisting motion and obtain the target meshes. The motion lasts two seconds, while the forearm twists clockwise and then counterclockwise. We apply the proposed method to deform the template so as to fit the target meshes. We first estimate the global rotation of the template by comparing the markers on the template and target meshes. The temporal consistency is also guaranteed using the temporal regularization when estimating the global rotation.

**Figure 11:**
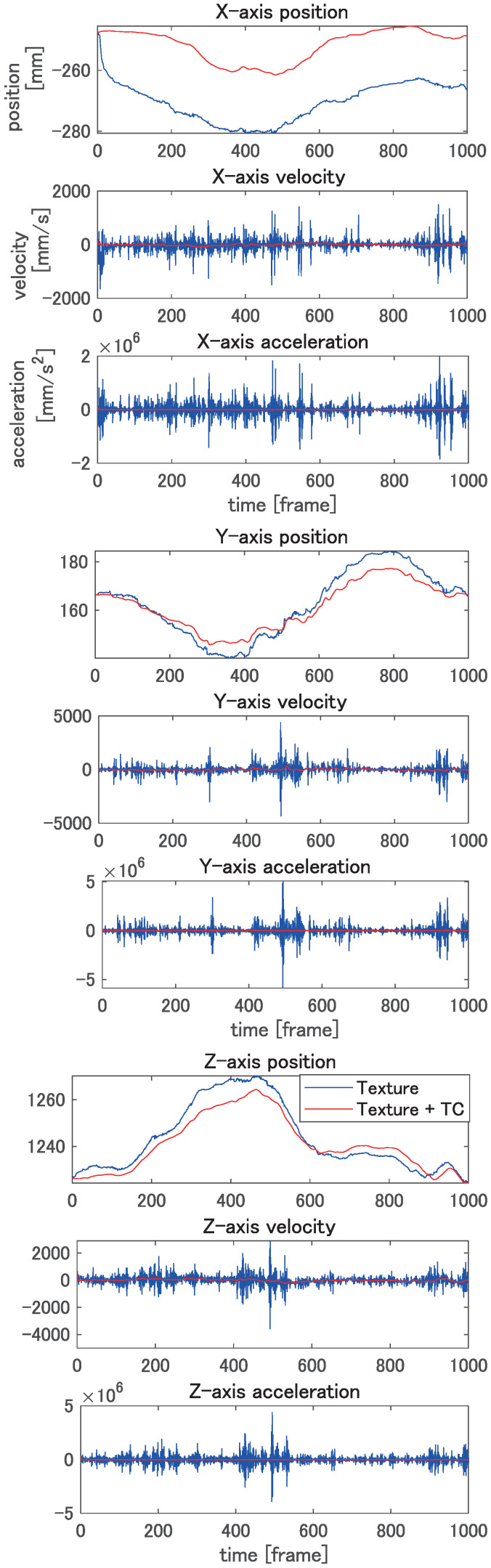
Results of arm twisting motion tracking. Upper row: positions, middle row: velocities, and lower row: acceleration in each axis.

By using the texture correspondence, the template can track the target meshes, and the arm twisting motion is successfully reconstructed as the result. Figure 11 shows the result of tracking the motion. Each column corresponds to a different axis, and rows correspond to the position, velocity, and acceleration.

In order to clarify the difference between the method with a TC and that without a TC, the obtained results are depicted by red and blue lines, respectively. As shown in Figure 11, the flicker noise along the trajectories is reduced. This effect is especially significant for velocities and accelerations. From this result, the proposed method can reconstruct not only the shape, but also the motion, including the velocity and acceleration. We can expect that the deformable registration can be applied to the motion analysis using the proposed method.

**Figure 12:**
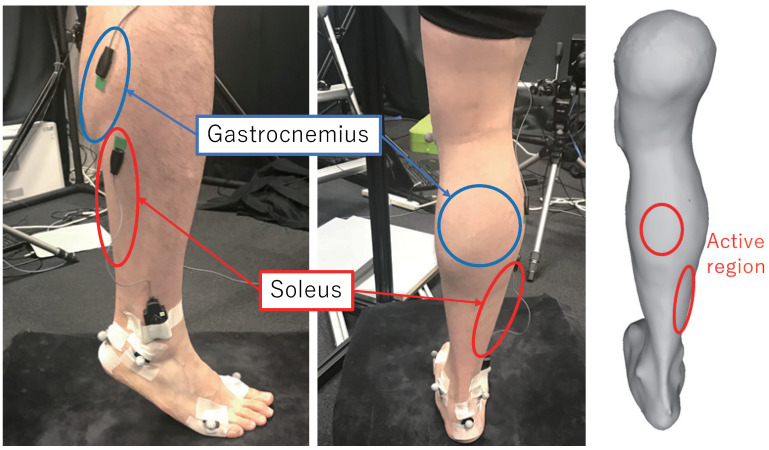
Electromyography sensor placement and results of 3D registration.

**Figure 13:**
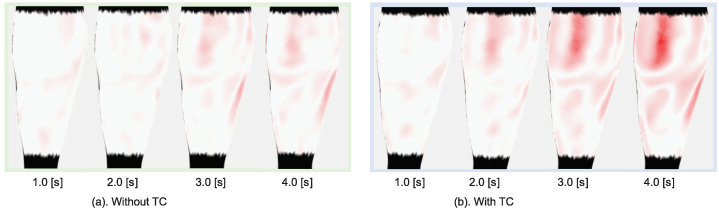
Heat map of strain from registration results.

**Figure 14:**
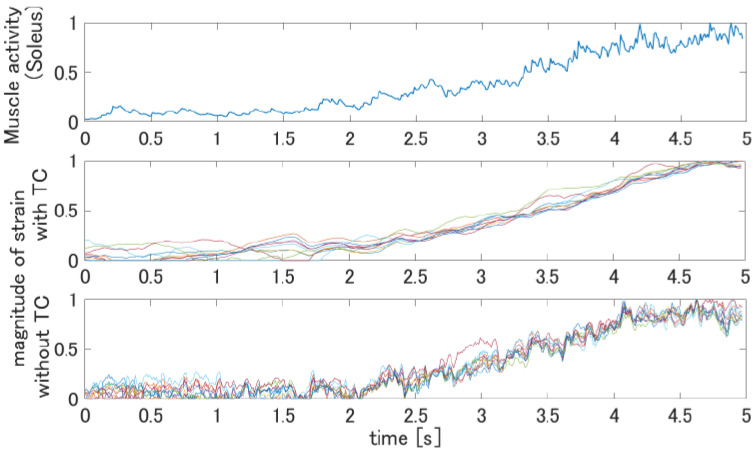
The blue line in the top graph is the measured muscle activity data, and the colored lines in the middle graph and the bottom graph show the magnitude of the strains in some vertices from muscle location.

We also apply the proposed method to the leg stretching motions that are captured and used in [1] to validate the feasibility of the muscle activity estimation by computing the skin strain. The motion lasts 5 s while the lower leg is stretching from the posture where the sole of the foot is on the floor to the posture of standing with the toe. Since the Gastrocnemius and Soleus muscles are expected to be active, as shown in Figure 12, we measure the EMG on these muscles to analyze the muscle activities. We follow the same procedure with the arm motion tracking but change the frame rate (100 FPS) of capturing the target meshes to synchronize the EMG measurements. By computing the strain tensor of the vertices in all frames, the heat map of the strain is obtained by following the same procedure. The results are shown in Figure 13. The right side of Figure 13a shows the heat map computed without temporal constraint and the left side (b) is with temporal constraints. Since the noises affect the computation of the strain tensor, the regions of the muscles cannot be observed in Figure 13a. On the other hand, the regions of the Gastrocnemius and Soleus muscles can be clearly seen in Figure 13b. We also show the muscle activity at the Gastrocremius muscle and the magnitude of the strain computed from the registration result with TC and without TC in Figure 14. As can be seen from Figure 14, there is a correspondence between the muscle activity and the magnitude of the strain.

### 4.2. Muscle Activity Prediction

For the experiment of muscle activity prediction, we used leg stretching motions that were captured and used in a previous study [1] and applied the proposed method to obtain the registration results.

**Figure 15:**
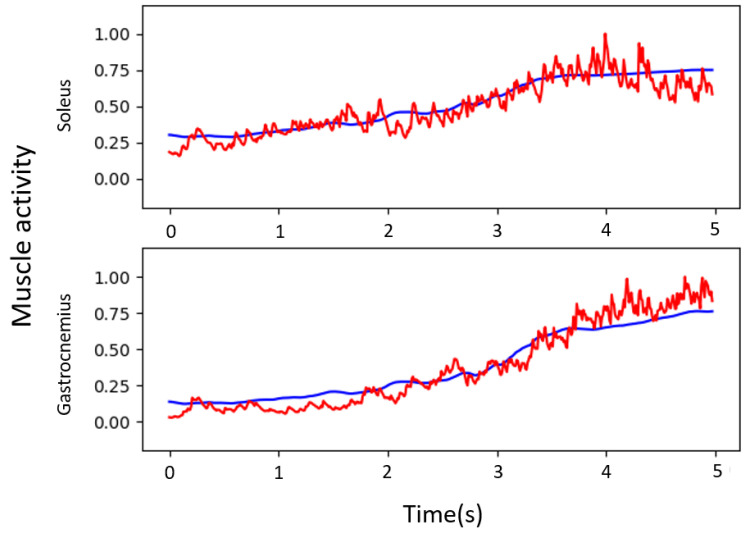
Prediction results for muscle activity. The red line indicates the results measured by EMG sensors, and the blue line indicates the prediction results.

The registration method of the present study is a template-based registration, so all groups of vertices of the action model possess the same index and nearest neighbor relationship after completing the registration. The original template has 15 k vertices, and, due to the missing top and bottom visual fields during data acquisition, we choose the strain information of all vertices of the mid-calf, where the data are more reliable, as training data. Each frame of the model used for training has a vertex count of 4.8 k. We use each motion as a batch, inputting all vertex strain information and outputting the muscle activity at two locations. Nine sets of actions are used as training data. Each action provides 498 frames of strain data. We train the network described in Section 3.4. The prediction results of the muscle activity are shown in Figure 15.

The muscle activity measured by the EMGs and the ones estimated by the proposed method were compared. The root mean squared errors (RMSEs) were 7.63% and 8.38% of the MVCs. Since the errors reported in [1] were 9.0% and 6.7%, the accuracy was comparable without the registration of bones, which are necessary in the method of [1]. In future work, we plan to improve them by modifying the network and using a large dataset.

## 5. Conclusions

In the present study, we proposed a method to predict human muscle activity from skin surface strain and used a non-rigid registration method with temporal constraint to accomplish better tracking of motion. For the registration, we introduce constraints on the time series in order to avoid the measurement noise in the vision system, which usually generates the displacement noise of the reconstructed shape. Otherwise, measurement noise will eventually lead to large noises in the velocities and accelerations on the point cloud, which affect our analysis of the dynamics of deformable objects. This process can be considered as a kind of filtering. In the current implementation, the temporal constraints are designed to regulate the velocities and accelerations to zero, and should be implemented as inequality constraints in the future. For the data analysis, we are currently computing the strains in the sense of statics. We should also compute the stress and the total energy, including the kinetic energy in a future study.

For muscle activity prediction, the proposed MLP network uses the dataset of surface strain information to predict muscle activity. We chose a simple lower limb case of a one-dimensional movement in order to ensure the same movement pattern and expect to use experimental data to infer a projection between muscle activity and skin surface strain of a certain fixed movement. This is limited by the fact that the mapping between EMG signals and muscle activity is not the same between movements. Since the training dataset is relatively small, the structure of the network is also quite simple. The prediction has strong limitations and fluctuations. In the future, we would like to optimize the network structure, collect data from more types of motion, and incorporate theoretical knowledge of human physiology in order to create a more reasonable predictive model.

## Figures and Tables

**Table 1:** Error of vertices position, velocity, and acceleration of registering the simulation model.

Position Error	x-axis	y-axis	z-axis
with TC	0.225	0.583	0.809
without TC	1.322	3.158	4.178
without TC + filter	0.921	1.450	1.627 mm
**Velocity Error**	**x-axis**	**y-axis**	**z-axis**
with TC	6.919	22.464	25.888
without TC	$0.928\times 10^{4}$	$4.061\times 10^{4}$	$5.972\times 10^{4}$
without TC + filter	$0.224\times 10^{3}$	$0.657\times 10^{3}$	$1.065\times 10^{3}$ mm/s
**Acceleration Error**	**x-axis**	**y-axis**	**z-axis**
with TC	$0.695\times 10^{3}$	$2.741\times 10^{3}$	$3.867\times 10^{3}$
without TC	$0.265\times 10^{9}$	$1.183\times 10^{9}$	$1.725\times 10^{9}$
without TC + filter	$0.449\times 10^{6}$	$1.430\times 10^{6}$	$2.321\times 10^{6}$ mm/s^2^

**Table 2:** Mean absolute percentage error of magnitude of strain from ground-truth.

Condition	MAPE of Strain
with TC	32.5
without TC	3946.9
without TC + filter	160.9%

## Data Availability

Not applicable.
